# Accuracy of intraocular lens power calculation formulae after laser refractive surgery in myopic eyes: a meta-analysis

**DOI:** 10.1186/s40662-020-00188-1

**Published:** 2020-05-01

**Authors:** Hongyu Li, Li Nan, Jun Li, Hui Song

**Affiliations:** 1 Clinical College of Ophthalmology, Tianjin Medical University, Tianjin, China; 2 Tianjin Eye Institute, Tianjin Key Lab of Ophthalmology and Visual Science, Tianjin Eye Hospital, No. 4 Gansu Road, He-ping District, Tianjin, 300020 China

**Keywords:** Myopic, Laser, Refractive, IOL power calculation, Meta-analysis

## Abstract

**Background:**

To compare the accuracy of intraocular lens power calculation formulae after laser refractive surgery in myopic eyes.

**Methods:**

We searched the databases on PubMed, EMBASE, Web of Science and the Cochrane library to select relevant studies published between Jan 1st, 2009 and Aug 11th, 2019. Primary outcomes were the percentages of refractive prediction error within ±0.5 D and ±1.0 D.

**Results:**

The final meta-analysis included 16 studies using seven common methods (ASCRS average, Barrett True-K no history, Double-K SRK/T, Haigis-L, OCT formula, Shammas-PL, and Wang-Koch-Maloney). ASCRS average yielded significantly higher percentage of refractive prediction error within ±0.5 D than Haigis-L, Shammas-PL and Wang-Koch-Maloney (*P* = 0.009, 0.01, 0.008, respectively). Barrett True-K no history also yielded significantly higher percentage of refractive prediction error within ±0.5 D than Shammas-PL and Wang-Koch-Maloney (*P* = 0.01, *P* < 0.0001, respectively), and a similar result was found when comparing OCT formula with Haigis-L and Shammas-PL (*P* = 0.03, *P* = 0.01, respectively).

**Conclusion:**

The ASCRS average or Barrett True-K no history should be used to calculate the intraocular lens power in eyes after myopic laser refractive surgery. The OCT formula if available, can also be a good alternative choice.

## Background

Patients who have had corneal excimer laser surgery are now facing cataract surgery with aging. It is a challenge for many ophthalmologists to accurately calculate the intraocular lens (IOL) power in eyes after refractive surgery. Calculating the IOL power using the third-generation formulae results in significant hyperopic error in eyes with previous myopic corneal refractive surgery [1]. For most myopic patients, the need for spectacles and hyperopia shift after cataract surgery are particularly bothersome. Two main sources of error in IOL power calculation after corneal refractive surgery exist: corneal power measurement error [2, 3] and effective lens position (ELP) error [4]. Error of corneal power is in itself a two-pronged problem. First, all manual and most topographers miss the central cornea which is the flattest area after myopic ablation. Second, the topographers do not consider the alteration of the ratio between anterior and posterior corneal curvature that occurs after excimer laser ablation, and thus use an inappropriate refractive index for corneal power calculation. The second main error is effective lens position. Although this is challenging in virgin eyes as well, it imposes additional challenge after laser ablation, especially in formulae that use corneal power, but not anterior chamber depth (ACD) to estimate ELP. All these errors lead to underestimation of IOL power in myopic refractive surgery and the opposite in hyperopic surgery.

Over the past few decades, various methods have been proposed to address the accuracy of IOL power calculation with patients after corneal refractive surgery. The clinical history method was once considered the gold standard for IOL power calculation in patients after refractive surgery. However, cataract surgeons still experience situations where historical data are not available or not credible. Therefore, the clinical history method was proved to be not as accurate as it was thought to be. Several formulae that exclude the dependence of historical data are available, including the Barrett True-K no history [5], Double-K method [6], Haigis-L [7], OCT formula [8], Shammas-PL [9], Wang-Koch-Maloney (W-K-M) [10], various IOL calculators [11], and others. Although the accuracy of these formulae is higher than the traditional formulae and the historical data method, the predictability amongst the abovementioned formulae is quite different in studies. Early studies demonstrated that the Haigis-L and Shammas-PL have good precision in IOL power calculation in eyes after corneal refractive surgery [12, 13]. Abulafia et al. [5] and Vrijman et al. [14] showed that the Barrett True-K no history was significantly more accurate than Haigis-L and Shammas-PL. Ianchulev et al. [15] suggested that the Barrett True-K no history produced a smaller percentage of refractive prediction error within ±0.5 D and ±1.0 D as compared with the Haigis-L. After Wang et al. provided the ASCRS calculator (available at: http://www.ascrs.org), the combined method became a good choice [16, 17]. Another new method i.e., the OCT formula, has been used in recent years. The debate about the best formula for IOL power calculation in eyes after laser refractive surgery still remains. The purpose of this meta-analysis was to compare the accuracy of IOL power calculation formulae without historical data in eyes after myopic laser refractive surgery.

## Methods

### Literature search

Two independent investigators (H.L. and L.N.) searched the databases of PubMed, EMBASE, Web of Science and the Cochrane library. We searched and selected relevant studies published between Jan 1st, 2009 and Aug 11th, 2019, using the following search terms for PubMed: (Lenses Intraocular [Mesh] OR Intraocular Lens [Title/Abstract] OR Implantable Contact Lens [Title/Abstract] OR IOL [Title/Abstract]) AND (Refractive Surgical Procedures [Mesh] OR Laser Corneal Surgeries [Title/Abstract] OR Laser Keratectomy [Title/Abstract] OR Laser Corneal Surgeries [Title/Abstract] OR Keratomileusis, Laser In Situ [Mesh] OR LASIK [Title/Abstract] OR Laser-Assisted Stromal In Situ Keratomileusis [Title/Abstract] OR Photorefractive Keratectomy [Mesh] OR PRK [Title/Abstract]) AND (calculate* OR formula*) AND (last 10 years [PDat]). Regardless of the primary outcome or language, we considered all possible studies for review. The two authors respectively evaluated the titles and abstracts of all searched studies and performed a manual search by searching the reference list of all the eligible articles.

### Inclusion and exclusion criteria

Inclusion criteria for studies were: (1) patients who had laser-assisted in situ keratomileusis (LASIK), photorefractive keratectomy (PRK) or laser-assisted subepithelial keratomileusis (LASEK) for myopia and subsequent uneventful cataract surgery; (2) at least two types of the following IOL power calculation formulae must be have been used: ASCRS average, Barrett True-K no history, Double-K SRK/T, Haigis-L, OCT formula, Shammas-PL, and W-K-M; (3) Optical biometry measured by partial coherence interferometry (PCI, IOL Master); (4) IOL constants were optimized.

Exclusion criteria for studies were: (1) patients who had hyperopic refractive surgery or radial keratotomy surgery; (2) percentage of refractive prediction error within ±0.5 D and ±1.0 D were unavailable; (3) eyes that have not in-the-bag fixed IOL implantation or another ocular surgery. Intraoperative refractive biometry [15], Shammas-PHL [18] and Olsen T formulae [19] were excluded because of their limited use in the clinic.

### Data extraction and quality assessment

We compared ASCRS average, Barrett True-K no history, Double-K SRK/T, Haigis-L, OCT formula, Shammas-PL, and W-K-M formulae which were used to calculate IOL power in eyes after myopic laser refractive surgery. The primary outcome assessed were as follows: the percentages of refractive prediction error within ±0.5 D and ±1.0 D. A higher percentage indicates higher precision of the formula. The two authors (H.L. and L.N.) independently extracted the data and compared the results. Discrepancies were resolved by another author (J.L.). We used a modified check-list adapted from the QUADAS-2 tool to assess the quality of the evidence [20, 21]. Study characteristics extracted from the retrieved studies were the first author, year of publication, sample size, the formula used and its percentages of refractive prediction error within ±0.5 D and ±1.0 D, and the postoperative refraction time and refraction method.

### Statistical analysis

The target outcome was the percentages of refractive prediction error within ±0.5 D and ±1.0 D of each formula. The refractive prediction error was calculated by subtracting the predicted spherical equivalent from the actual postoperative spherical equivalent. For categorical outcomes, we calculated pooled estimates of the odds ratio (OR) with a fixed-effects model. Studies included in the same meta-analysis are different, which is called heterogeneity. The I^2^ statistic was used to determine heterogeneity across studies, such that heterogeneity was quantified irrespective of the number of studies. It could describe the percentages of heterogeneity caused by each study rather than sampling error. An I^2^ value greater than 50% was considered as substantial heterogeneity. We also conducted sensitivity analysis and subgroup analysis to evaluate the change in overall effect when the I^2^ value was greater than 50%. Funnel plots were used to evaluate publication bias and small-study effect. Data pooling was done by using Review Manager (version 5.3, Cochrane Collaboration, Oxford, UK). A *P* value less than 0.05 was considered to be statistically significant.

## Results

**Fig. 1:**
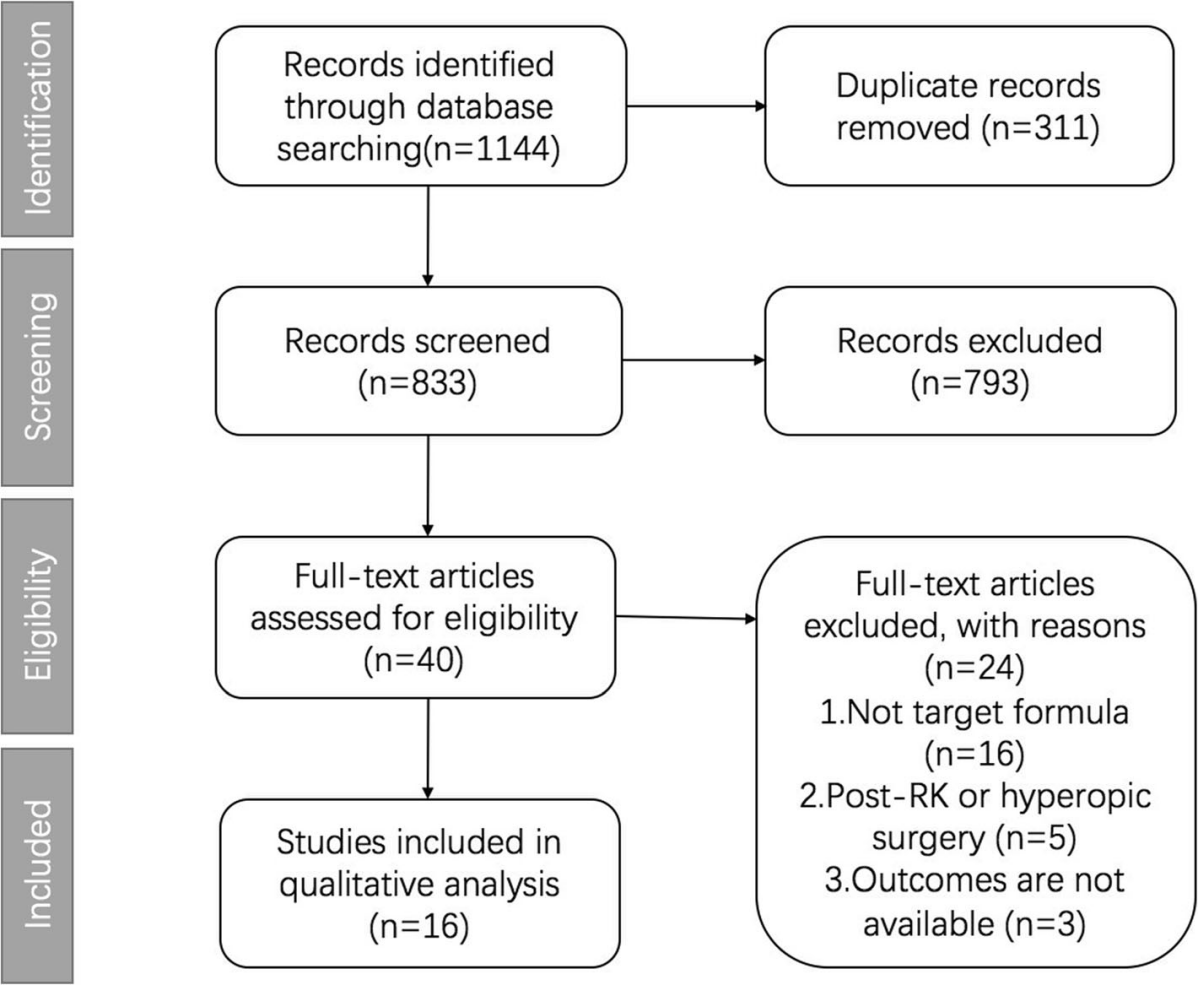
Flowchart of articles selection (RK = radial keratotomy)

A total of 1144 articles were initially identified by literature search (Fig. 1). Among them, after removal of duplicates, 833 articles remained, of which 793 records were removed due to irrelevance. The remaining 40 articles were chosen for full-text evaluation. Among these, three studies did not have percentage data, 16 studies included only one of the selected formulae, five studies were hyperopic laser refractive surgery or radial keratotomy surgery. After the exclusion of these studies, 16 articles were used for analysis [5, 12–17, 22–30].

### Study characteristics and quality assessment

**Fig. 2:**
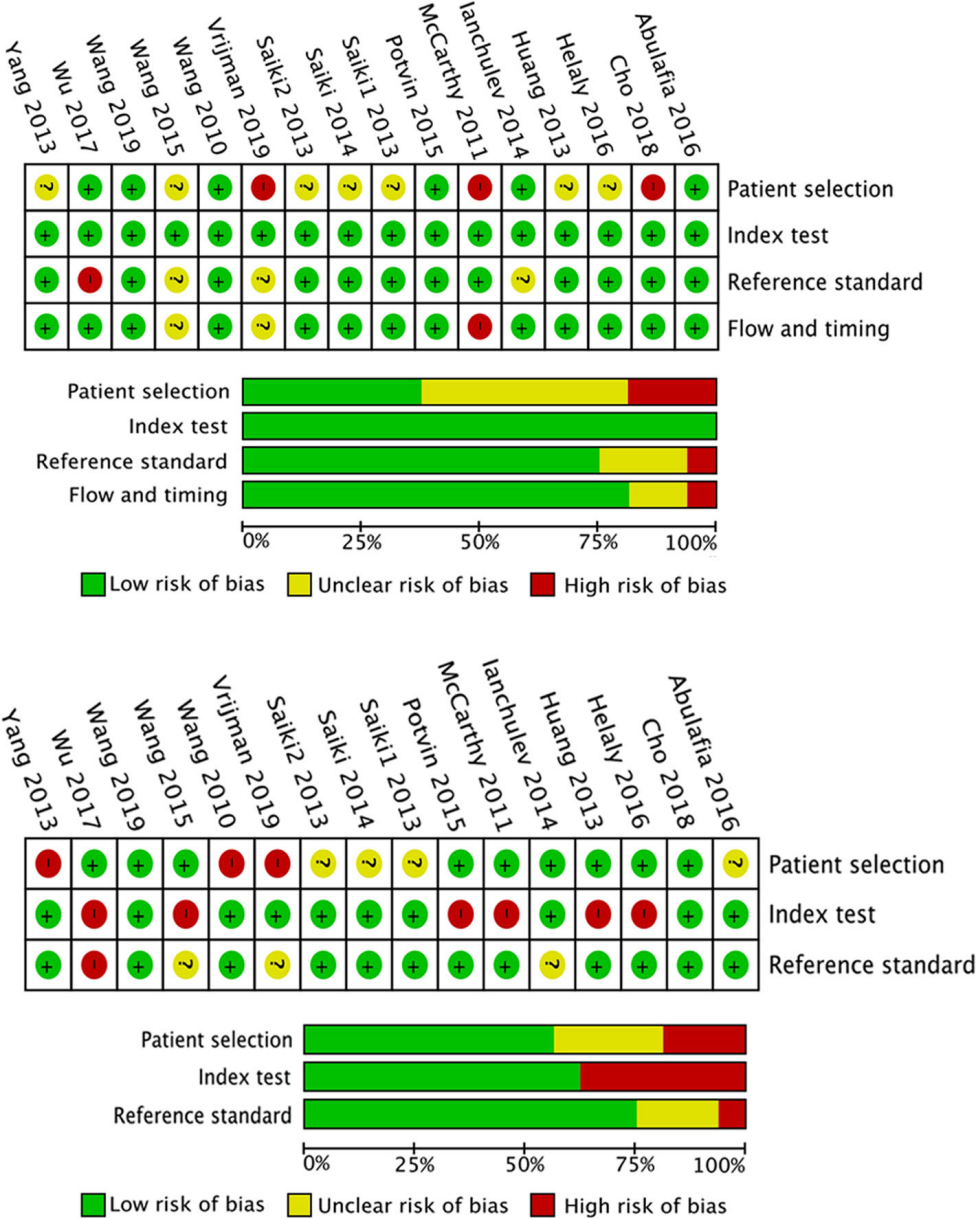
Quality assessment of the eligible studies according to the modified QUADAS-2

In total, there were 1167 eyes enrolled in the 16 studies (Table 1). Most of the studies (*n* = 15) included patients implanting a mono-focal IOL in the capsular bag, only one study had multifocal IOL implantation, and another included patient with unclear exclusion. The quality assessment included in the study was performed using the modified QUADAS-2 (Fig. 2). Additional file 1 provides detailed information on the comprehensive assessment. For patient selection, three studies had inappropriate exclusions, resulting in a high risk of bias. Seven studies did not clarify patient enrollment methods, resulting in an unclear risk of bias. For reference standard and flow assessment, one study performed subjective refraction and its follow-up time was less than 3 weeks. For the index test, all sixteen studies were of high quality.

**Table 1:** Characteristics of study participants

Author	Year	Eyes	Age (years, mean ± SD)	AL (mm, mean ± SD)	Follow-up (days)	Refraction	HL	SHL	BTK	DK	WKM	ASCRS	OCT
Abulafia [5]	2016	58	NA	25.85 ± 1.35	> 21	objective	√	√	√		√	√	
McCarthy [12]	2011	173	57.0 ± 0.0	26.9 ± 1.86	203	objective	√	√		√			
Wang [13]	2010	72	58.0 ± 8.0	26.19 ± 1.55	> 21	objective	√	√			√	√	
Vrijman [14]	2019	64	NA	25.28 ± 1.4	NA	NA	√	√	√			√	
Ianchulev [15]	2014	246	NA	25.43 ± 1.43	30–90	NA	√	√					
Yang [16]	2013	62	61.0 ± 6.79	25.98 ± 1.55	90–180	objective	√	√			√	√	
Wang [17]	2015	104	63.0 ± 7.0	25.46 ± 1.3	21–90	objective	√	√	√		√		√
Huang [22]	2013	46	61.5 ± 8.0	NA	> 30	objective	√	√					√
Saiki1 [23]	2013	25	54.0 ± 9.9	26.39 ± 0.99	> 30	objective	√	√		√			
Saiki2 [24]	2013	28	54.0 ± 9.8	26.19 ± 1.06	> 30	objective	√	√		√			
Saiki [25]	2014	24	54.0 ± 10.6	NA	> 30	objective	√	√		√			
Potvin [26]	2015	101	NA	25.83 ± 1.36	NA	NA	√	√			√		
Helaly [27]	2016	45	51.27 ± 7.31	28.66 ± 2.78	30–120	objective	√	√		√			
Wu [28]	2017	10	50.3 ± 9.0	30.06 ± 2.87	> 90	subjective	√	√					
Cho [29]	2018	56	54.6 ± 9.37	27.04 ± 2.36	90	objective	√	√	√		√		
Wang [30]	2019	53	64.5 ± 7.1	25.72 ± 1.64	> 21	objective	√		√				

*AL=* axial length, *HL=* Haigis-L, *SHL=* Shammas-PL, *BTK=* Barrett true K no history, *DK=* Double-K SRK/T, *WKM=* Wang-Koch-Maloney, *ASCRS=* ASCRS average, *OCT=* optical coherence tomography formula, *NA=* not available

### Outcomes

**Fig. 3:**
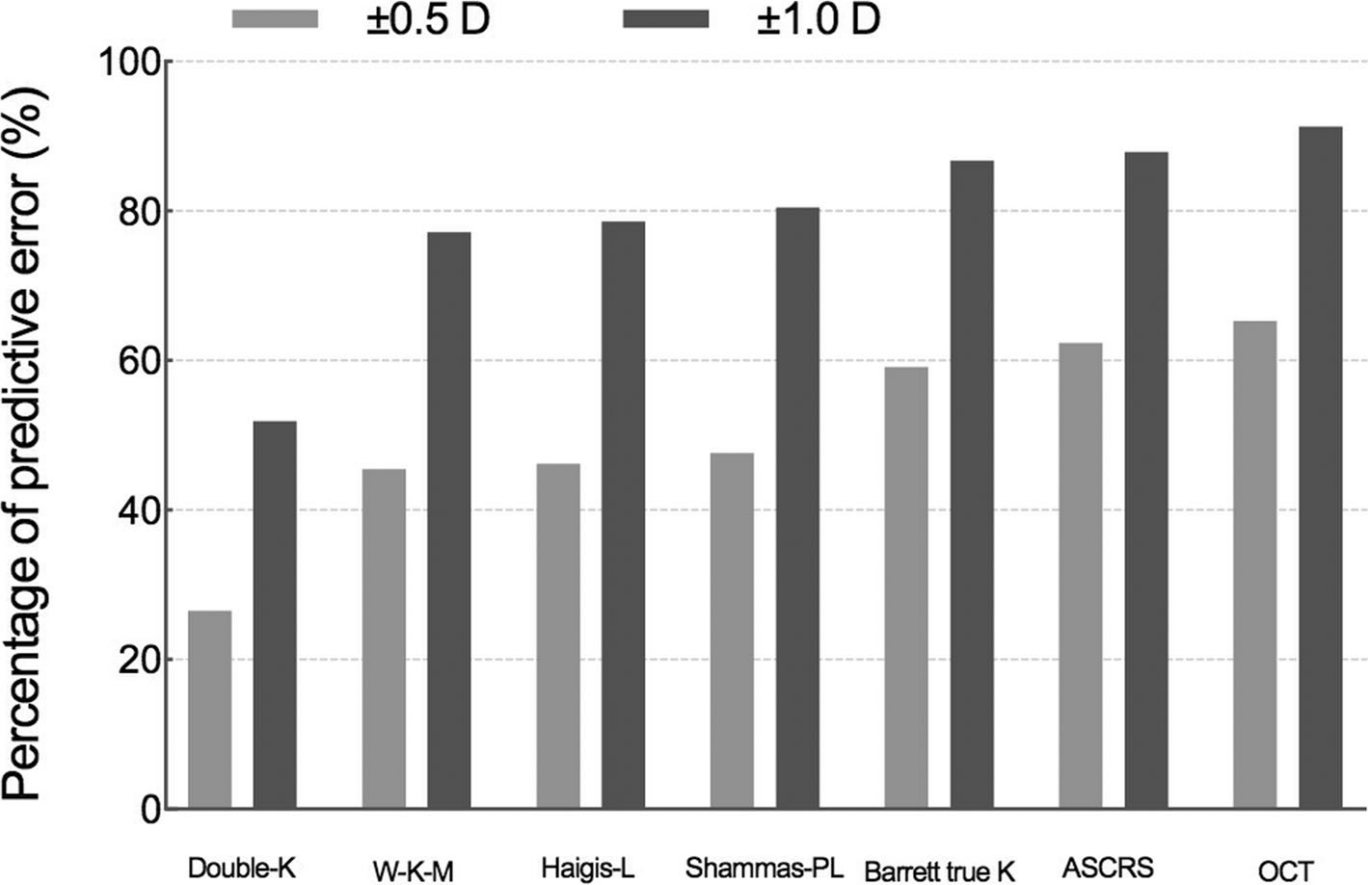
The overall percentage of refractive prediction error within ±0.5 D and ±1.0 D of the included formulae. ASCRS average means average degree from ASCRS calculator; Barrett True-K means Barrett True-K no history; Double-K means Double-K SRK/T; OCT means optical coherence tomography formula; W-K-M means Wang-Koch-Maloney; D means diopter

Among the 1167 eyes enrolled, 332 eyes were calculated with ASCRS average, 279 with Barrett True-K no history, 291 with Double-K SRK/T, 1019 with Haigis-L, 150 with OCT formula, 1055 with Shammas-PL, and 433 with W-K-M. The overall percentages of refractive prediction error within ±0.5 D (±1.0 D) of the above formulae are 62.35% (87.95%), 59.14% (86.74%), 26.46% (51.89%), 46.22% (78.61%), 65.33% (91.33%), 47.68% (80.47%), and 45.50% (77.14%), respectively (Fig. 3).

### Percentage of refractive prediction error within ±0.5 D

**Fig. 4:**
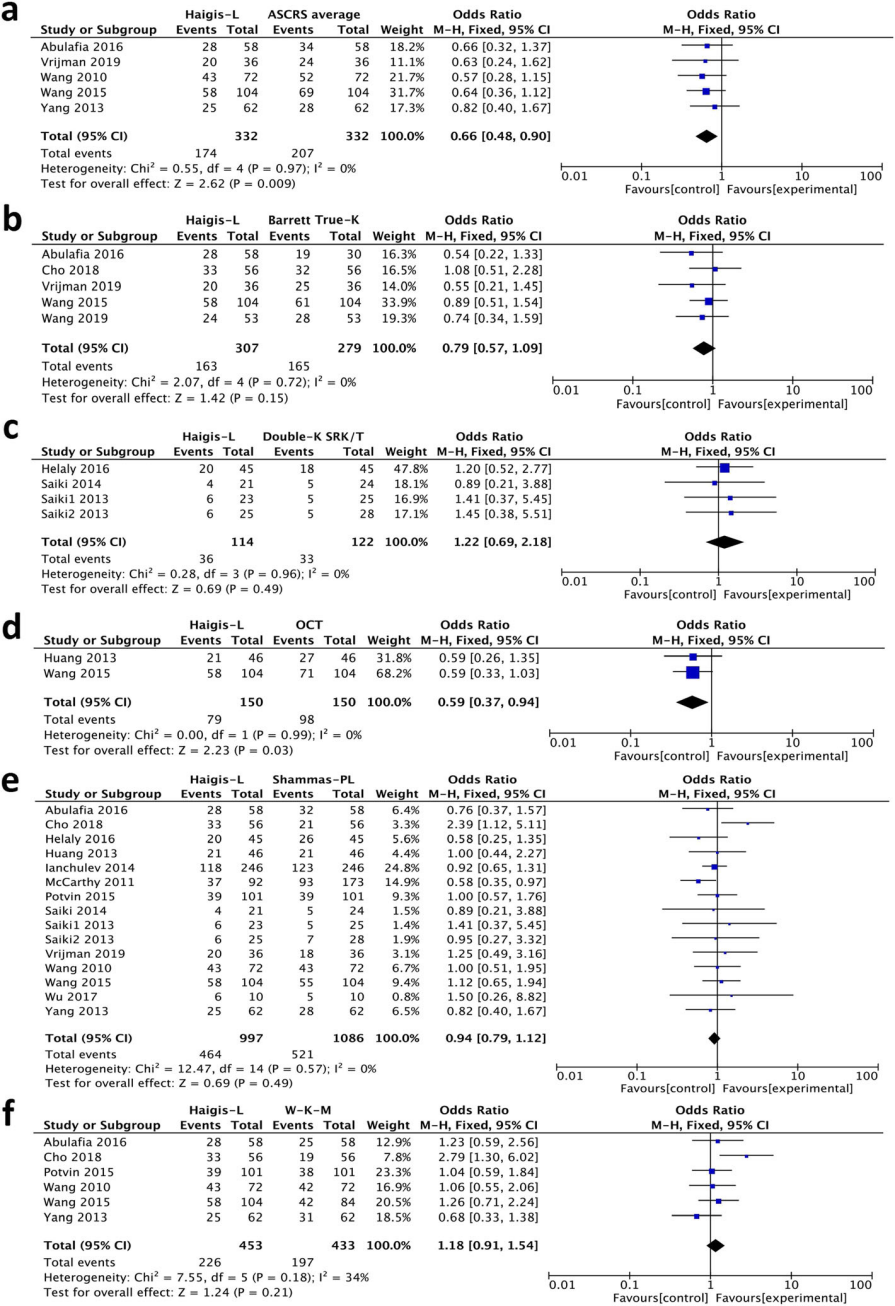
Forest plots comparing the percentage of refractive prediction error within ±0.5 D between Haigis-L and ASCRS average (**a**) Barrett True-K no history (**b**), Double-K SRK/T (**c**), OCT formula (**d**), Shammas-PL (**e**), and W-K-M (**f**) (Note: Barrett True-K means Barrett True-K no history)

Figure 4 shows the comparisons of the percentage of refractive prediction error within ±0.5 D between Haigis-L and the other formulae. The percentage of refractive prediction error within ±0.5 D calculated by the Haigis-L was significantly lower than the ASCRS average (Fig. 4a, *P* = 0.009) and OCT formula (Fig. 4d, *P* = 0.03). Shammas-PL also produced significantly lower percentages than ASCRS average, Barrett True-K no history, and OCT formula (Additional file 2, *P* = 0.01, *P* = 0.01, *P* = 0.01, respectively). The percentages obtained by ASCRS average and Barrett True-K no history was significantly higher than W-K-M (Additional file 2, *P* < 0.0001 and *P* = 0.008, respectively).

### Percentage of refractive prediction error within ±1.0 D

**Fig. 5:**
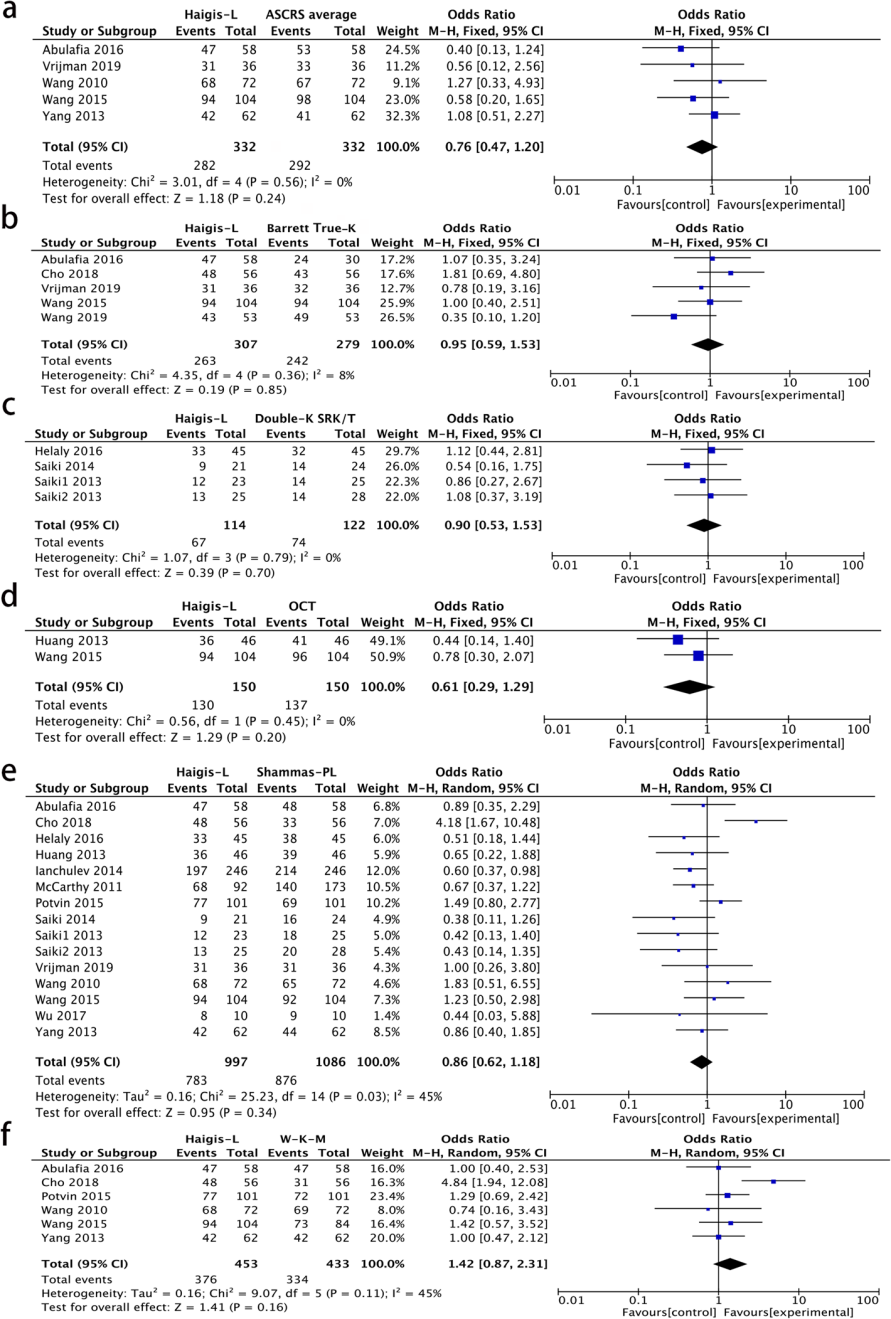
Forest plots comparing the percentages of refractive prediction error within ±1.0 D between Haigis-L and ASCRS average (**a**) Barrett True-K no history (**b**), Double-K SRK/T (**c**), OCT formula (**d**), Shammas-PL (**e**), and W-K-M (**f**) (Note: Barrett True-K means Barrett True-K no history)

Figure 5 shows the comparisons of the percentage of refractive prediction error within ±1.0 D between Haigis-L and other formulae. No significant statistical difference was found when comparing Haigis-L with the other formulae (Additional file 3).

### Heterogeneity and subgroup analysis

The I^2^ values and 95% confidence interval (CI) are shown in Figs. 4 and 5. Using pairwise comparison, substantial heterogeneity was detected in two pairs and the random-effect model was applied. The sensitivity analysis showed that by omitting Cho 2018, I^2^ significantly decreased to 0% in the comparison of the percentage of refractive prediction error within ±1.0 D between Haigis-L and Shammas-PL and between Haigis-L and W-K-M (Additional file 4, *P* = 0.0004, *P* = 0.004, respectively). Cho et al. (2018) did not show details of the IOL constant optimization procedure and did not report whether the keratometry was measured from the IOL Master. After omitting this study, the I^2^ value decreased. There was no significant finding from the funnel plot (Additional file 5).

## Discussion

Calculating the IOL power in eyes after refractive surgery is still a challenging task for most ophthalmologists. Chen et al. [31] have done a meta-analysis of evaluating the accuracy of IOL power calculation methods after laser refractive surgery in myopic eyes and found that many methods without historical data had similar accuracy compared with Haigis-L. However, they did not include the new formulae without historical data, such as ASCRS average, Barrett True-K no history or OCT formula. To our knowledge, this is the first meta-analysis to assess the accuracy of the different IOL power calculation formulae with no historical data in myopic eyes after laser refractive surgery by measuring the percentage of refractive prediction error within ±0.5 D and ±1.0 D.

In order to control heterogeneity and biasness, we excluded the studies which performed optical biometric measurement by using devices such as ultrasound biometry (UB) or corneal topography, and which did not perform the constant optimization procedure. Previous studies have shown that the refractive prediction errors of the patients after cataract surgery were attributed to axial length (AL) measurement (54%), post-ACD estimate (38%), and keratometry measurement (8%). Roy et al. [32] indicated that the IOL Master had more accurate AL measurement than UB and the former was the gold standard for optical biometric measurement [33]. Besides, due to the use of different types of IOLs, it was essential for each formula to use a constant optimization procedure to equate the mean error to zero.

Our meta-analysis suggests that only the overall percentages of refractive prediction error within ±0.5 D or ±1.0 D of ASCRS average, Barrett True-K no history or OCT formula can reach the benchmark standard of 55% being within ±0.5 D and 85% within ±1.0 D of refractive prediction error for virgin eyes after phacoemulsification [34]. In the percentage of refractive prediction error within ±0.5 D analysis: ASCRS average was more accurate than the Haigis-L, Shammas-PL and W-K-M methods. It was not surprising because the ASCRS calculator produced an average IOL power (ASCRS average) and became a good choice for surgeons, especially for those who cannot choose a proper calculation method or judge which method was the best for their patients [16, 17]. In addition, our results also indicated that Barrett True-K no history was more accurate than Shammas-PL and W-K-M but not Haigis-L. Barrett True-K no history is based on Barrett universal II formula, which was first proposed in 2009 [5], however the mathematical formula was not published. It could be used for free on the following websites (http://www.apacrs.org or http://www.ascrs.org), and studies have found that they lead to accurate refractive results and is now considered as one of the most reliable options after myopic and hyperopic LASIK/PRK [15, 35]. Another formula is Shammas-PL [9] which used data available at the time of cataract surgery to predict the post refractive surgery keratometry and W-K-M [13] which converted anterior corneal power from Atlas topography, both of which are regression formulae. Haigis-L, based on the regular Haigis formula, which uses three constants (a_0_, a_1_, a_2_) to predict the ELP, has proven to avoid error in corneal power measurement [7]. In recent years, Haigis-L became the most popular method for IOL power calculation after refractive surgery, especially for Asians and Germans [31, 36]. It is easily available on the IOL Master. No statistical differences between Haigis-L and Shammas-PL and W-K-M were found in our study, which is in tandem with previous reports [12].

We also included the Double-K method in this meta-analysis, which was elaborated by Aramberri et al. [6]: using keratometry of pre-refractive surgery which could be replaced as 43.5D or 43.86D to estimate the ELP and keratometry post-refractive surgery for IOL power calculation. The Double-K method can be combined with different formulae, such as SRK/T, Hoffer Q, and Holladay II. In our meta-analysis, even though Double-K SRK/T had the lowest total percentage of refractive prediction error within ±0.5 D and ±1.0 D, there was no statistically significant difference between Double-K SRK/T, Haigis-L, and Shammas-PL. One reason might be the limited sample sizes of Double-K SRK/T (291 eyes) vs. Haigis-L (1019 eyes) and Shammas-PL (1055 eyes). Another reason might be the different keratometry of pre-refractive surgery that was used in the enrolled studies. We suggest that if the Haigis-L or Shammas-PL could be obtained, these formulae would be better than the Double-K method. Previous studies have shown that the Double-K method had good predictability of IOL power calculation when refractive historical data was known, but its accuracy decreased when historical data was unknown [17, 29]. In addition, when we included Cho et al.’s 2018 study, I^2^ was 54% and there was no significant difference between Haigis-L and Shammas-PL (*P* = 0.34, Fig. 5e). However, when we omit Cho et al’s 2018 study, I^2^ reduced to 0% (*P* = 0.02, Additional file 4A). There actually was no statistically significant difference between these two formulae in our study because the diamond intersects the vertical line in Fig. 5e. Further studies need to be conducted to confirm this result.

Another latest IOL power calculation method, OCT formula, has the highest value of overall percentages which were 65.33% within ±0.5 D and 91.33% within ±1.0 D in our analysis. Compared to other types of corneal tomography, the faster speed of corneal mapping and higher axial resolution of OCT gave cataract surgeons an alternative choice to measure both the anterior and posterior corneal power [8]. OCT had good repeatability and accuracy in measuring total corneal power even in the presence of corneal opacities [37, 38]. This formula was based on the paraxial approximation of Gaussian optics, and used ACD, AL and lens thickness (LT) to predict the ELP [39]. Only a few studies have reported and compared its accuracy. Huang et al. [22] found that OCT formula had a significantly lower mean absolute error (MAE) than Haigis-L and Shammas-PL. Wang et al. [17] indicated that the OCT formula had a higher percentage of prediction refractive error within ±0.5 D and ±1.0 D than ASCRS average and Barrett True-K no history. In our meta-analysis, OCT formula which included 150 eyes, had a better accuracy than the Haigis-L and Shammas-PL, but no statistical difference was found when comparing the OCT formula with ASCRS average and Barrett True-K no history. However, due to the limited number of eyes in the eligible articles, further studies should be made to confirm our findings.

This meta-analysis has several limitations. Firstly, several studies were retrospective case series with a limited sample size and there was also a bias caused by the variability of patient characteristics, IOL types and single-center analysis. However, we chose to accept this limitation when comparing the accuracy of IOL power calculation formulae. Next, optical biometric data of all eligibility studies were measured using PCI. As the popularity of the Scheimpflug imaging use in eyes after refractive surgery is increasing, the precision of various formulae with optical biometry needs to be further confirmed. Finally, we did not include hyperopic refractive surgery eyes and the other recent formulae (intraoperative refractive biometry, Shammas-PHL, Olsen) because of the limited number of studies. Few studies have evaluated the accuracy of IOL power calculation in patients after hyperopic LASIK or PRK. According to recent reports [14, 40, 41], IOL power calculation methods using no previous data or using the change in manifest refraction were not significantly different. In addition, the Barrett True-K no history formula was not superior compared with other formulae. More studies are needed to explore the accuracy of different IOL power calculation formulae after refractive surgery in hyperopic eyes.

## Conclusions

Our meta-analysis indicates that the application of ASCRS average, Barrett True-K no history or OCT formula in the eyes after refractive surgery are promising with the higher percentage of eyes within ±0.5 D of prediction error when compared to Shammas-PL and W-K-M. ASCRS average and OCT formula also have higher percentages compared with the Haigis-L. We suggest that the ASCRS average or Barrett True-K no history should be used to calculate the IOL power in eyes after myopic laser refractive surgery. If the surgeon is able to use the OCT formula, it can also be a good alternative choice.

## Supplementary information

**Additional file 1.** Modified check-list adapted from the QUADAS-2 tool.

**Additional file 2.** Pairwise comparisons between formulae within ±0.5 D.

**Additional file 3.** Pairwise comparisons between formulae within ±1.0 D.

**Additional file 4.** Subgroups analysis.

**Additional file 5.** Funnel plot of the percentages within ±0.5 D.

## Data Availability

The datasets supporting the conclusions of this article are included within the article and its additional file.

## Abbreviations

ACD: Anterior chamber depth
AL: Axial length
ASCRS: American Society of Cataract and Refractive Surgery
CI: Confidence interval
D: Diopter
ELP: Effective lens position
IOL: Intraocular lens
LASEK: Laser-assisted subepithelial keratomileusis
LASIK: Laser-assisted in situ keratomileusis
LT: Lens thickness
MAE: Mean absolute error
OCT: Optical coherence tomography
OR: Odds ratio
PCI: Partial coherence interferometry
PRK: Photorefractive keratectomy
UB: Ultrasound biometry
W-K-M: Wang-Koch-Maloney
