# Supplementary material for: Accuracy of intraocular lens power calculation formulae after laser refractive surgery in myopic eyes: a meta-analysis
Source: Eye Vis (Lond). 2020 May 1;7:37. doi: 10.1186/s40662-020-00188-1 (PMC7339492; doi:10.1186/s40662-020-00188-1)
Supplement: Supplementary file 1 — Additional file 1. Modified check-list adapted from the QUADAS-2 tool. [file 40662_2020_188_MOESM1_ESM.pdf]

Appendix 1. Modified check-list adapted from the QUADAS-2 tool

Assessment of risk of bias

Domain 1: patient selection

Question 1: Was a consecutive or random sample of patients enrolled?

- • 'yes' → low risk of bias
- • 'unclear' → unclear risk of bias
- • 'no' → high risk of bias.

Question 2: Did the study avoid inappropriate exclusions?

- • 'no' for < 10% of patients or 'yes' → low risk of bias
- • 'unclear' → unclear risk of bias
- • 'no' for ≥ 10% of patients → high risk of bias.

Domain 2: index test

Were the index test result read without knowing the result of the reference standard?

- • 'yes' → low risk of bias
- • 'unclear' → unclear risk of bias
- • 'no' → high risk of bias.

Domain 3: reference standard

The method in testing the postoperative refraction

- • 'objective autorefraction' → low risk of bias
- • 'unclear' → unclear risk of bias
- • 'subjective refraction' → high risk of bias.

Domain 4: flow and timing

Question 1: Were all patients included in the analysis?

- • 'no' but for < 10% of patients or 'yes' → low risk of bias
- • 'unclear' → unclear risk of bias

- • ‘no’ for ≥ 10% of patients → high risk of bias.

Question 2: Was there appropriate interval between the surgery and reference standard?

- • three weeks after surgery or later → low risk of bias
- • ‘unclear’ → unclear risk of bias
- • before three weeks postoperatively → high risk of bias.

Assessment of applicability concerns

Domain 1: patient selection

Was a different diagnosis presented?

- • ‘no’ → low risk of bias
- • ‘unclear’ → unclear risk of bias
- • ‘yes’ → high risk of bias.

Domain 2: index test

Variations in test technology

- • ‘no’ → low risk of bias
- • ‘unclear’ → unclear risk of bias
- • ‘yes’ → high risk of bias.

Domain 3: reference standard

The method in testing the postoperative refraction

- • ‘objective autorefraction’ → low risk of bias
- • ‘unclear’ → unclear risk of bias
- • ‘subjective refraction’ → high risk of bias.

Author/Year	Risk of Bias				Applicability Concerns		
	patient selection	index test	reference standard	flow and timing	patient selection	index test	reference standard
Wang 2010	L	L	L	L	H	L	L
McCarthy 2011	H	L	L	U	L	H	L
Huang 2013	U	L	L	L	L	H	L
Saiki1 2013	U	L	L	L	H	L	L
Saiki2 2013	U	L	L	L	H	L	L
Yang 2013	U	L	L	L	H	L	L
Ianchulev 2014	L	L	U	L	L	L	U
Saiki 2014	U	L	L	L	L	H	L
Potvin 2015	L	L	L	L	L	H	L
Wang 2015	U	L	U	U	L	H	U
Abulafia 2016	L	L	L	L	U	L	L
Helaly 2016	U	L	L	L	L	H	L
Wu 2017	L	L	H	L	L	H	H
Cho 2018	U	L	L	L	L	L	L
Vrijman 2019	H	L	U	U	H	L	U
Wang 2019	L	L	L	L	L	L	L

L: low risk; H: high risk; U: unclear risk
