# Supplementary figures and images for: Accuracy of intraocular lens power calculation formulae after laser refractive surgery in myopic eyes: a meta-analysis
Source: Eye Vis (Lond). 2020 May 1;7:37. doi: 10.1186/s40662-020-00188-1 (PMC7339492; doi:10.1186/s40662-020-00188-1)

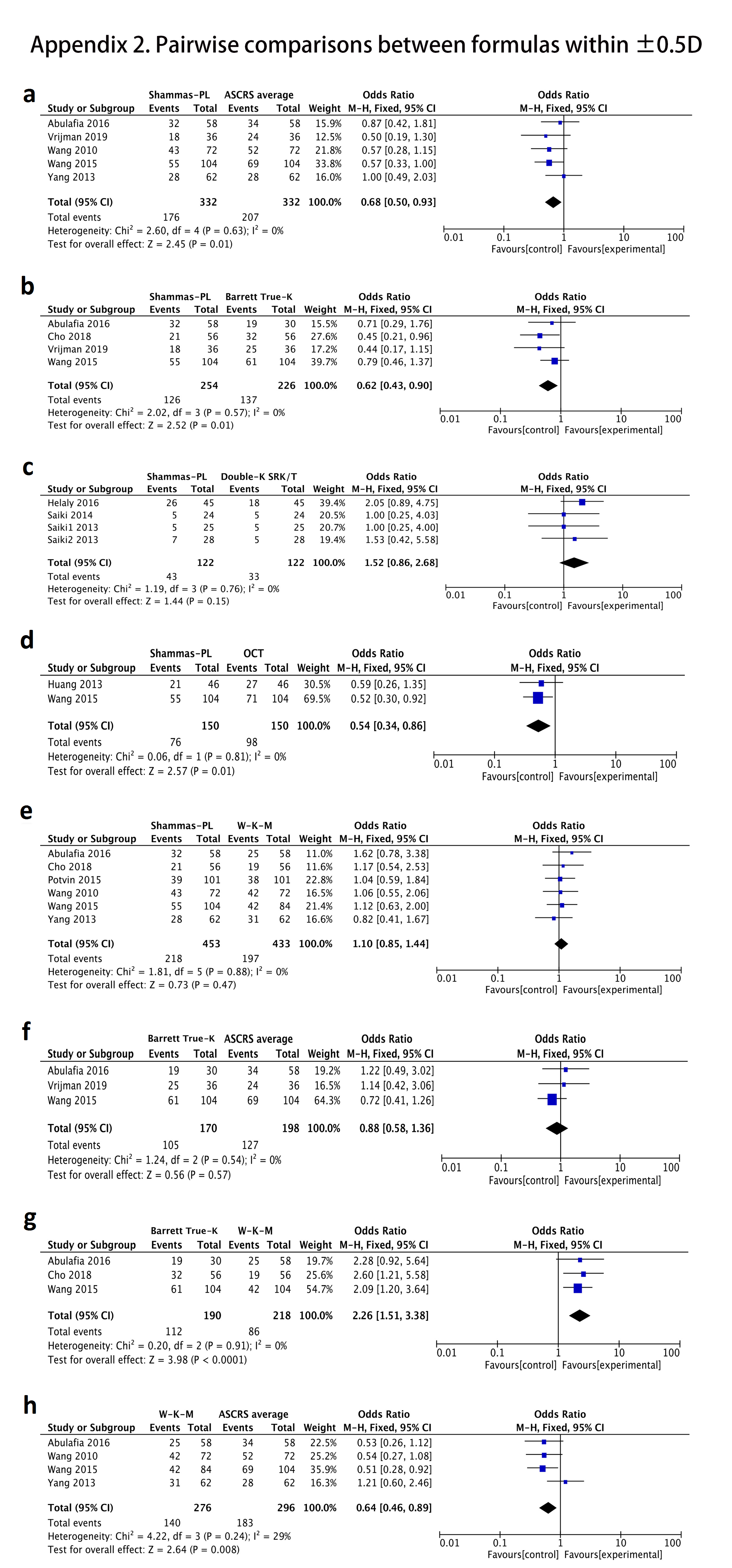

Supplement: Supplementary file 2 — Additional file 2. Pairwise comparisons between formulae within ±0.5 D. [file 40662_2020_188_MOESM2_ESM.tif]

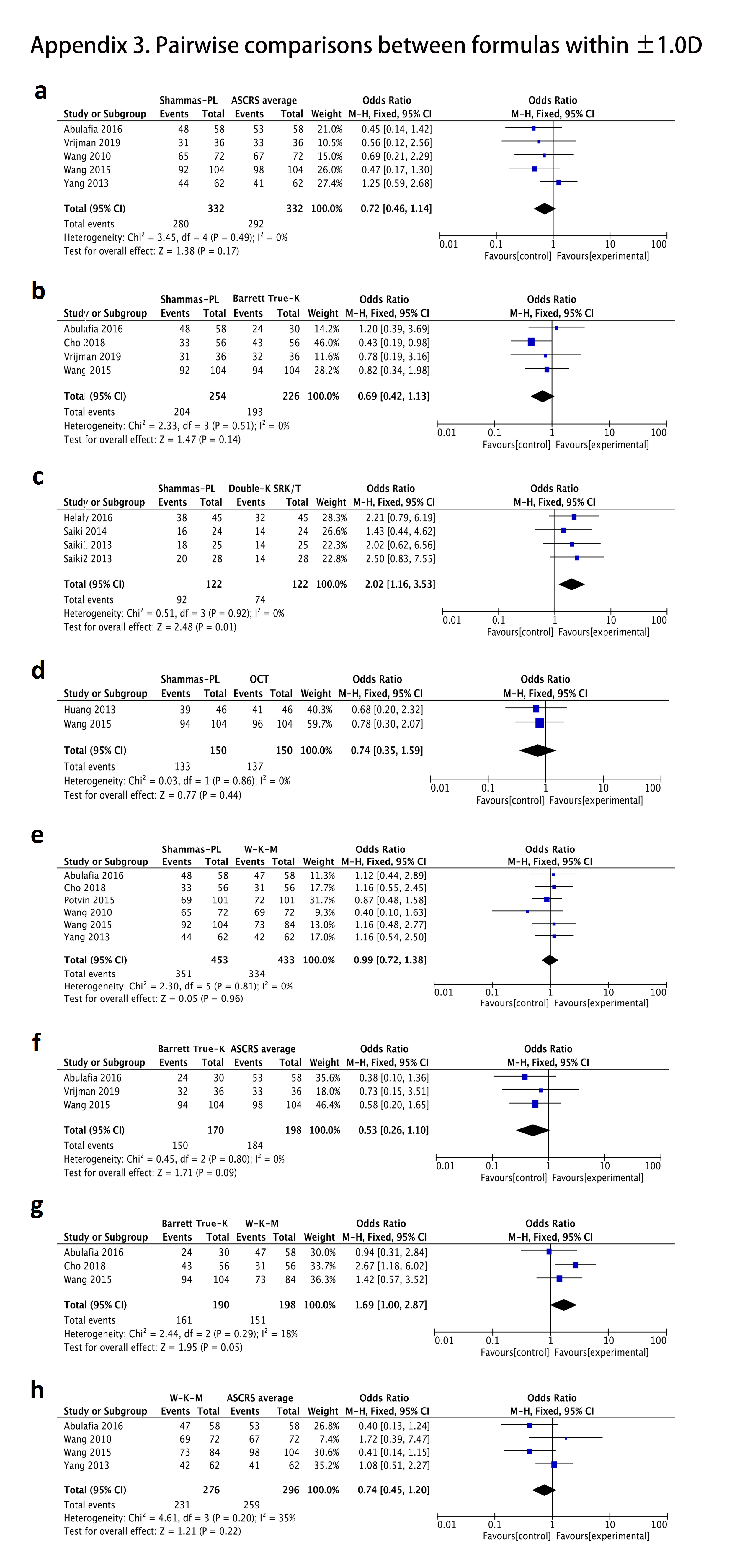

Supplement: Supplementary file 3 — Additional file 3. Pairwise comparisons between formulae within ±1.0 D. [file 40662_2020_188_MOESM3_ESM.tif]

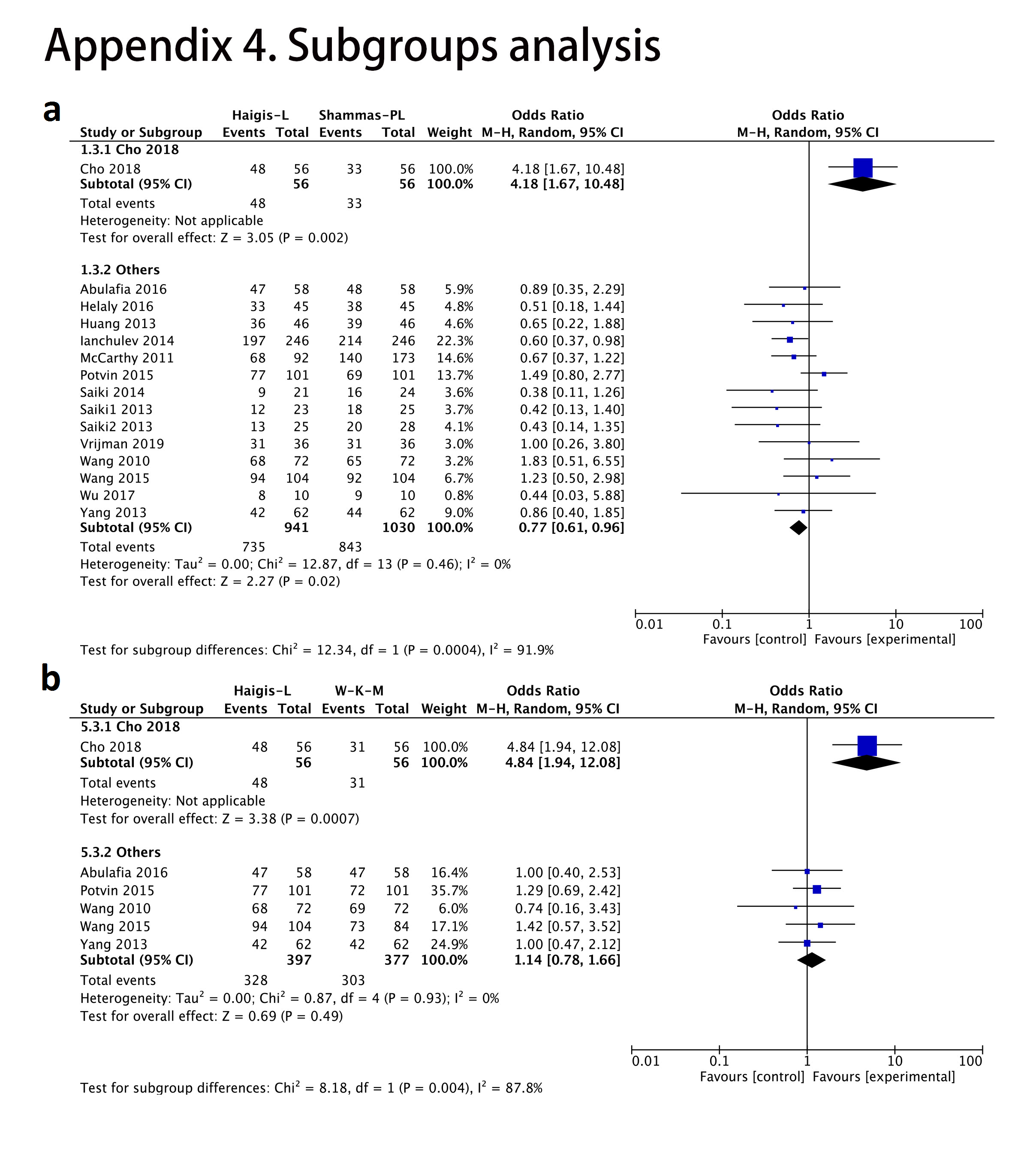

Supplement: Supplementary file 4 — Additional file 4. Subgroups analysis. [file 40662_2020_188_MOESM4_ESM.tif]

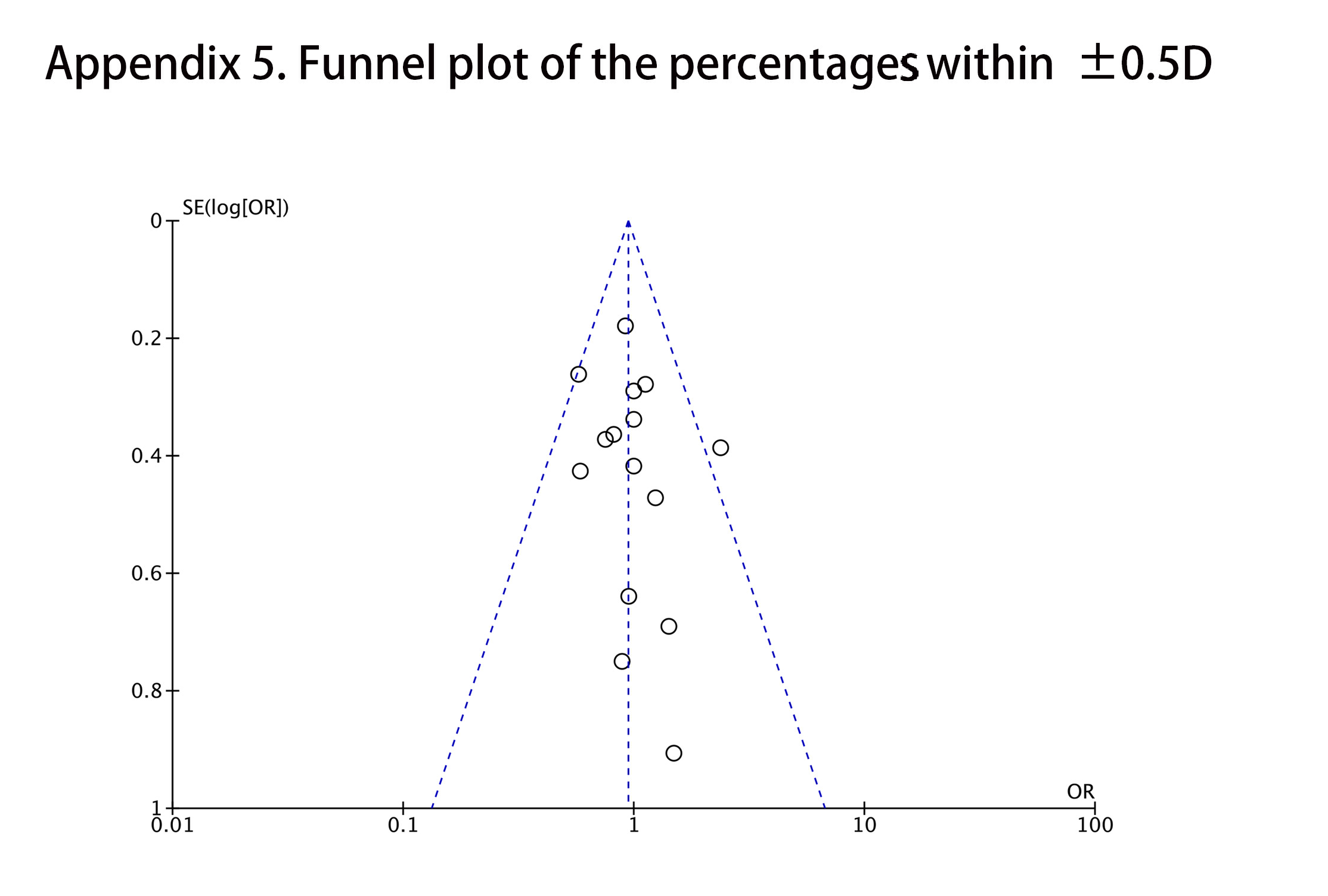

Supplement: Supplementary file 5 — Additional file 5. Funnel plot of the percentages within ±0.5 D. [file 40662_2020_188_MOESM5_ESM.tif]
